# Relationship Between Loneliness and Hypothyroidism

**DOI:** 10.7759/cureus.19955

**Published:** 2021-11-28

**Authors:** Khalid Alshehri, Nada E Algethami, Rahma A Algethami, Raghad H ALAyyubi, Ghaida H Alotaibi, Jawaher S Alotaibi, Sheikha A Altawairqi

**Affiliations:** 1 Family Medicine, Prince Mansour Military Hospital, Taif, SAU; 2 Medicine, College of Medicine, Taif University, Taif, SAU

**Keywords:** thyroid hormones, saudi arabia, hypothyroid patients, loneliness predictors, r-ucla questionnaire

## Abstract

Aim

This study aimed to assess the relationship between loneliness and hypothyroidism in patients with hypothyroidism and to determine predictors of loneliness.

Materials and methods

A cross-sectional study was conducted on hypothyroid patients attending the endocrine clinics at Alhada Military Hospital and Prince Mansour Military Hospital, Taif, Saudi Arabia between the period of December 2020 and May 2021. Patients with more than 18 years of hypothyroidism were included and those with other thyroid diseases were excluded as well as those with other comorbidities and psychiatric disorders and those who were living alone. Data were collected using an online pre-structured questionnaire.

Results

The study included 231 hypothyroid patients with a mean age of 43.34 ± 12.9 years, and 90.9% were females. The majority (96.5%) were taking levothyroxine, and 27.3% were practicing physical activity. Only 2.2% of the participants had a high degree of loneliness, whereas 47.2%, 34.6%, and 16% had low, moderate, and moderately high degrees of loneliness, respectively.

Discussion

In this study, 2.2% of sampled hypothyroid patients had high (2.2%) or moderately high degrees of loneliness feelings (16%). Duration of hypothyroidism was a significant predictor for high loneliness score.

## Introduction

Thyroid hormones have a major effect on all major systems, and adequate levels for optimal function are essential [1]. Pathological processes in patients with thyroid diseases commonly arise from the thyroid gland and are presented by conditions associated with deficiency of the thyroid hormones (hypothyroidism) or excessive release of hormones (hyperthyroidism) [2]. Clinical thyroid dysfunction presentation is non-specific; therefore, the diagnosis of thyroid dysfunction is based primarily on biochemical confirmation [3]. Moreover, the most sensitive marker of thyroid dysfunction is the thyroid-stimulating hormone (TSH) level [4].

Hypothyroidism is common throughout the world. Previous studies conducted in different regions in Saudi Arabia have shown a high prevalence of hypothyroidism [3,4]. In primary hypothyroidism, the disease is in the thyroid gland itself, and TSH concentrations will be above the reference range with free T4 levels below the reference range, whereas secondary hypothyroidism is due to an anterior pituitary gland defect, resulting in decreased secretion of TSH [1,5].

The relationship between thyroid hormones and TSH is influenced by many variables, including smoking, age, and thyroid peroxidase antibody status [6]. In adults, the major symptoms of hypothyroidism include cold intolerance, dry skin, and changes in voice pattern, constipation, lethargy, fatigue, and mood disorders [7,8].

Loneliness is defined as emotional distress that is characterized by feelings of sadness due to the lack or absence of predictable meaningful social relationships [9]. One study performed in the United States showed that the prevalence of loneliness was 16.9%. However, the risk of loneliness increases with non-married status, poor self-esteem, lower educational level, and chronic diseases [10]. In addition, for sociodemographic characteristics, authors have reported that loneliness is more common in women than men [11]. Loneliness has a negative impact or influence on multiple chronic diseases [12].

Furthermore, loneliness has been related to the development of chronic diseases. One study explored the associations between loneliness on metabolic biomarkers and inflammatory dysregulation. It was found that loneliness has an impact on metabolic biomarkers [13].

Social-contextual feelings, such as loneliness, affect the thyroid functioning and adaptive immune system. One study showed more fluctuation in TH secretion among people who scored low in terms of social support. This might also indicate that people who receive social support have relatively stable TH secretion [14]. In a study performed in 1999, they studied the difference between healthy and chronically ill people and found greater feelings of loneliness in ill people (in terms of their social network) [15]. Adequate social support has an association with better health and lowers loneliness among people [16].

Moreover, we cannot ignore the health impacts of loneliness among the elderly [17]. Despite the improved health consequences, the prevalence rates have persisted over several decades [18]. Only a few reviewers have focused on them despite being the highest group who experience loneliness [19]. Others have acknowledged the significant impact of loneliness on elderly people because of their normal aging process and the presence of comorbidity diseases [20]. The main effect is on their chronic diseases, which do not improve, and it becomes the main source of morbidity and mortality among the elderly [18].

To the best of our knowledge, there are very few studies that have been performed to assess the relationship between loneliness and hypothyroidism. This study aimed to determine the associations between loneliness and hypothyroidism and correlates loneliness among hypothyroid patients.

## Materials and methods

Study design, setting, and time frame

This was a cross-sectional study conducted at the endocrine clinics at Alhada Military Hospital and Prince Mansour Military Hospital, Taif, Saudi Arabia between the period of December 2020 and May 2021. The research was approved by the Research Ethics Committee of Armed Forces Hospitals, Taif region (2020-515).

Study participants

Patients who were more than 18 years of age with hypothyroidism (TSH less than 0.3500mIU/L) were included, and we excluded those with other thyroid diseases, other comorbidities, psychiatric disorders, and who are living alone.

Data collection

Data were collected through phone interviews using the valid online revised University of California, Los Angeles (R-UCLA) Questionnaire (Appendix Table 4). The questionnaire included the following items: personal data, such as marital status, age, gender, level of education, and socioeconomic status, and clinical data, such as duration of hypothyroidism and medication dose. Lab data were collected from medical records, including the levels of TSH, CBC, vitamin B12, and vitamin D.

Clinical measurements

To screen for loneliness, the validated R-UCLA Questionnaire was used [1]. The R-UCLA includes 20 items with each one containing a 4-point Likert scale. The maximum overall score for the 20 items is 80, and the minimum score is 20. Scores between 65 and 80 indicate a high degree of loneliness, 50 to 64 indicate a moderately high degree of loneliness, 35-49 indicate a moderate degree of loneliness, and 20-34 indicate a low degree of loneliness, according to Perry’s loneliness classification [21,22].

Data analysis 

Data were analyzed using the SPSS program version 26 (IBM Corp., Armonk, NY). Qualitative data were expressed as numbers and percentages, and the Chi-squared test (χ^2^) was used to test the relationship between variables. Quantitative data were presented as mean and standard deviation (mean ± SD) and the one-way ANOVA test and Kruskal Wallis tests were used according to the data normality. Correlation analysis using the Spearman’s and Pearson correlation tests was done according to the data normality. A p-value of less than 0.05 was considered statistically significant.

## Results

Table 1 shows that the mean of the participants was 43.34 ± 12.9 years, 90.9% were females, 84.4% were married and 40.3% had a high secondary school level of education or less. Most of them (63.6%) had a medium socioeconomic status (SES), 1.3% were current smokers, and most of them were obese (39.8%). The majority (96.5%) were taking levothyroxine and 27.3% were practicing physical activity.

**Table 1:** Distribution of the participants according to their characters, smoking, BMI, levothyroxine use, and physical activity SES: socioeconomic status, TSH: thyroid-stimulating hormone, MCHC: mean corpuscular hemoglobin concentration, MCH: mean corpuscular hemoglobin, MCV: mean corpuscular volume

Variable	No. (%)
Age (mean ± SD)	43.34 ± 12.9
Gender	
Male	21 (9.1)
Female	210 (90.9)
Marital status	
Married	195 (84.4)
Single	25 (10.8)
Divorced	5 (2.2)
Widowed	6 (2.6)
Level of education	
High secondary school or less	93 (40.3)
Graduated school	28 (12.1)
Bachelor’s degree	25 (10.8)
Post-graduate education (Master or above)	44 (19)
Illiterate	41 (17.7)
SES	
Low (less than 5000 SR)	52 (22.5)
Medium (5000–15,000)	147 (63.6)
High (more than 15,000 SR)	32 (13.9)
Smoking	
Yes	3 (1.3)
No	201 (87)
Ex-smoker	7 (3)
Passive smoker	20 (8.7)
BMI categories	
Underweight	9 (3.9)
Normal weight	51 (22.1)
Overweight	79 (34.2)
Obese	92 (39.8)
Levothyroxine use	
No	8 (3.5)
Yes	223 (96.5)
Physical activity	
Yes	63 (27.3)
No sedentary lifestyle	168 (72.7)
If physically active, frequency of exercise	
No activity	168 (72.7)
<150 min/week	35 (15.2)
150–300 min/week	17 (7.4)
>300 min/week	11 (4.8)
Variable	(Mean ± SD)
Smoking duration for current smokers year	10.7 ± 13.57
Hypothyroidism duration/years	8.97± 5.79
BMI (kg/m^2^)	29.23 ± 8.11
Levothyroxine dose	86.65 ± 42.36
TSH	3.76 ± 5.67
T4	12.93 ± 2.71
T3	5.33 ± 10.31
MCHC	129.2 ± 138.23
MCV	83.24 ± 8.99
MCH	27.06 ± 3
Hb	97.28 ± 53.21
Vit B12	323.84 ± 148.6
Vit D	28.82 ± 24.75
Score	37.58 ± 11.41

The mean smoking and hypothyroidism duration were 10.7 ± 13.57 and 8.97± 5.79 years and the mean BMI was 29.23 ± 8.11 kg/m^2^, respectively. The mean levels of levothyroxine dose, TSH, T4, and T3 levels were 86.65 ± 42.36, 3.76 ± 5.67, 12.93 ± 2.71, and 5.33 ± 10.31, respectively. Mean levels of MCHC, MCV, MCH, Hb, Vit B12 and Vit D were 129.2 ± 138.23, 83.24 ± 8.99, 27.06 ± 3, 97.28 ± 53.21, 323.84 ± 148.6 and 28.82 ± 24.75, respectively. The mean score was 37.58 ± 11.41 (Table 2).

**Table 2:** Relationship between the degree of loneliness and participants’ characters, smoking, BMI, levothyroxine use, physical activity, and quantitative variables (patients’ s characters and clinical data) N.B.:* Kruskal Wallis test; **one-way ANOVA test SES: socioeconomic status, TSH: thyroid-stimulating hormone, MCHC: mean corpuscular hemoglobin concentration, MCH: mean corpuscular hemoglobin, MCV: mean corpuscular volume

Variable	Degree of loneliness				Chi-squared test	P-value
	Low no. (%)	Moderate no. (%)	Moderately high no. (%)	High no. (%)		
Gender					3.64	0.303
Male	8 (38.1)	6 (28.6)	6 (28.6)	1 (4.8)		
Female	101 (48.1)	74 (35.2)	31 (14.8)	4 (1.9)		
Marital status					4.06	0.907
Married	89 (45.6)	70 (35.9)	31 (15.9)	5 (2.6)		
Single	13 (52)	7 (28)	5 (20)	0 (0.0)		
Divorced	4 (80)	1 (20)	0 (0.0)	0 (0.0)		
Widowed	3 (50)	2 (33.3)	1 (16.7)	0 (0.0)		
Level of education					8.9	0.711
High secondary school or less	40 (43)	33 (35.5)	16 (17.2)	4 (4.3)		
Graduated school	13 (46.4)	10 (35.7)	4 (14.3)	1 (3.6)		
Bachelor’s degree	14 (56)	6 (24)	5 (20)	0 (0.0)		
Post-graduate education (Master or above)	24 (54.5)	13 (29.5)	7 (15.9)	0 (0.0)		
Illiterate	18 (43.9)	18 (43.9)	5 (12.2)	0 (0.0)		
SES					5.14	0.525
Low (less than 5000 SR)	23 (44.2)	18 (34.6)	11 (21.2)	0 (0.0)		
Medium (5000–15000)	68 (46.3)	53 (36.1)	21 (14.3)	5 (3.4)		
High (more than 15,000 SR)	18 (56.3)	9 (28.1)	5 (15.6)	0 (0.0)		
Smoking					3.79	0.924
Yes	1 (33.3)	1 (33.3)	1 (33.3)	0 (0.0)		
No	95 (47.3)	70 (34.8)	32 (15.9)	4 (2)		
Ex-smoker	5 (71.4)	1 (14.3)	1 (15.3)	0 (0.0)		
Passive smoker	8 (40)	8 (40)	3 (15)	1 (5)		
BMI categories						
Underweight	5 (55.6)	4 (44.4)	0 (0.0)	0 (0.0)		
Normal weight	25 (49)	15 (29.4)	9 (17.6)	2 (3.9)		
Overweight	37 (46.8)	31 (39.2)	11 (13.9)	0 (0.0)		
Obese	42 (45.7)	30 (32.6)	17 (18.5)	3 (3.3)		
Levothyroxine use					2.02	0.567
No	2 (25)	4 (50)	2 (25)	0 (0.0)		
Yes	107 (48)	76 (34.1)	35 (15.7)	5 (2.2)		
Physical activity					1.09	0.778
Yes	30 (47.6)	23 (36.5)	8 (12.7)	2 (3.2)		
No sedentary lifestyle	79 (47)	57 (33.9)	29 (17.3)	3 (1.8)		
If physically active, frequency of exercise					14.42	0.108
No activity	79 (47)	57 (33.9)	29 (17.3)	3 (1.8)		
<150 min\week	18 (51.4)	14 (40)	2 (5.7)	1 (2.9)		
150-300 min\week	11 (64.7)	4 (23.5)	2 (11.8)	0 (0.0)		
>300 min\week	1 (9.1)	5 (45.5)	4 (36.4)	1 (9.1)		
Age (mean ± SD)	43.07 ± 13.77	42.91 ± 11.63	44.11± 12.52	50 ± 17.26	3*	0.731
Hypothyroidism duration/years (mean ± SD)	7.79 ± 4.6	10.46 ± 6.99	8.86 ± 5.58	11.6 ± 4.21	3*	0.028
BMI (kg/m^2^; mean ± SD)	29.11 ± 7.74	29.25 ± 9.64	29.4 ± 5.64	30.21 ± 6.47	3*	0.77
Levothyroxine dose (mean ± SD)	85.61 ± 39.82	84.01 ± 39.7	95 ± 54.72	87.5 ± 32.27	3*	0.798
TSH (mean ± SD)	3.52 ± 4.02	3.58 ± 4.74	4.87 ± 9.88	2.51 ± 1.91	3*	0.91
T4 (mean ± SD)	13.26 ± 3.08	12.73 ± 2.17	12.6 ± 2.26	12.12 ± 2.09	3*	0.64
T3 (mean ± SD)	3.86 ± 0.68	7.7 ± 16.73	4.01± 0.93	5.2 ± 0.83	2*	0.165
MCHC (mean ± SD)	116.36 ± 133.9	149.06 ± 142.92	128.45 ± 142.24	101.63 ± 137.58	3*	0.148
MCV (mean ± SD)	83.32 ± 7.7	82.25 ± 10.96	85.11 ± 8.21	84.5 ± 5.3	3*	0.397
MCH (mean ± SD)	26.96 ± 2.84	26.97 ± 3.18	27.55 ± 3.22	27.38 ± 2.87	3*	0.619
Hb (mean ± SD)	99.38 ± 52.11	92.04 ± 55.84	102.29 ± 51.63	94.5 ± 61.02	3*	0.782
Vit B12 (mean ± SD)	362.44 ± 97.44	326.52 ± 182.88	242.33 ± 102.78	420 ± 120.45	0.94**	0.434
Vit D (mean ± SD)	27.47 ± 19.36	30.53 ± 27.31	24.01 ± 19.37	56.7 ± 67.31	3*	0.183

**Figure 1:**
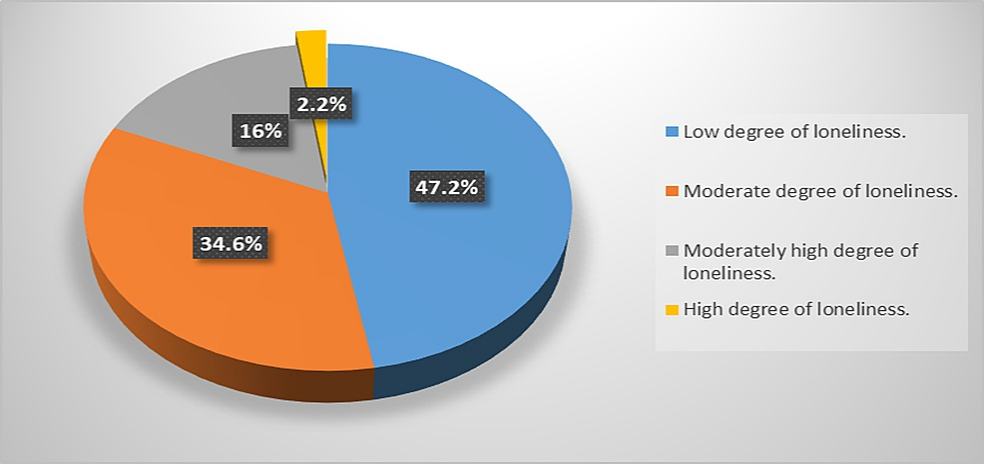
Relationship between the degree of loneliness and participants’ characters, smoking, BMI, levothyroxine use, physical activity, and quantitative variables (patients’ characters and clinical data)

Figure 1 illustrated that only 2.2% of the participants had a high degree of loneliness, while 47.2%, 34.6%, and 16% had low, moderate, and moderately high degrees of loneliness, respectively.

Table 3 and Appendix Table 4 demonstrated that participants who had a high degree of loneliness had a significantly higher mean hypothyroidism duration (p ≤ 0.05). On the other hand, a non-significant relationship was found between the degree of loneliness and participants’ gender, marital status, educational level, SES, smoking, BMI, levothyroxine use, physical activity, or clinical data other than hypothyroidism duration (p ≥ 0.05).

**Table 3:** Spearman’s correlation analysis between R-UCLA scores and participants’ characters and clinical data N.B.:*Pearson correlation test R-UCLA score: revised University of California, Los Angeles score, MCHC: mean corpuscular hemoglobin concentration, MCH: mean corpuscular hemoglobin, MCV: mean corpuscular volume

Variable	R-UCLA score	
	r	p-value
Age (mean SD)	0.009	0.887
Smoking duration (for current smokers)/years	0.008	0.231
BMI (kg/ m^2^)	0.02	0.673
Levothyroxine dose	0.04	0.513
TSH	−0.001	0.987
T4	−0.07	0.33
T3	0.24	0.055
MCHC	0.05	0.468
MCV	0.07	0.28
MCH	0.05	0.406
Hb	0.005	0.947
Vit B12	−0.18*	0.312
Vit D	0.06	0.406

Table 3 shows that a non-significant positive correlation was present between the R-UCLA scores and age, smoking duration, BMI, levothyroxine dose, T3, MCHC, MCV, MCH, Hb, or Vit D levels (p ≥ 0.05). At the same time, a non-significant negative correlation was present between the R-UCLA scores and TSH and Vit B12 levels (p ≥ 0.05).

**Figure 2:**
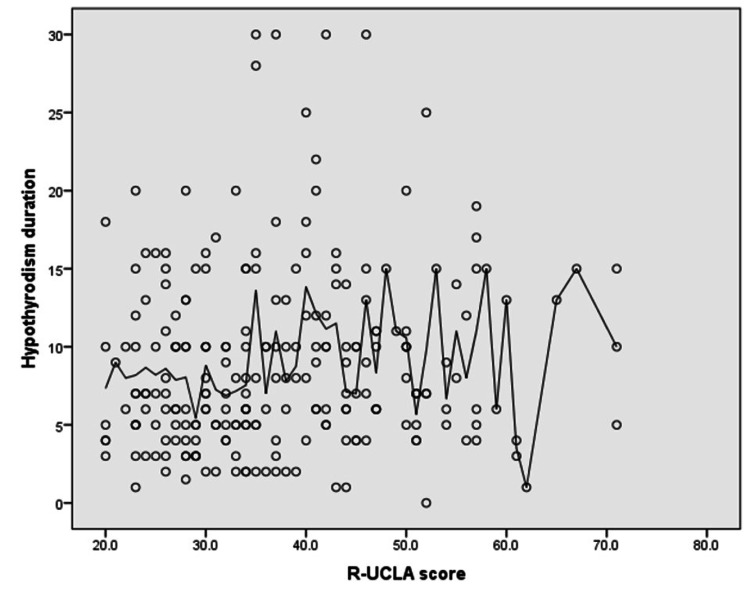
Spearman’s correlation analysis between R-UCLA scores and hypothyroidism duration/years R-UCLA scores: revised University of California, Los Angeles scores

Figure 2 shows that a significant positive correlation was found between the R-UCLA scores and hypothyroidism duration/years (r = 0.12, p-value = 0.035).

## Discussion

The current study aimed to assess the relationship between loneliness and hypothyroidism in patients with hypothyroidism and to determine predictors of loneliness level. Depression with a poor mood is one of the reported effects of clinical hypothyroidism, and that intimately reflects the mood-related symptoms of people with a depressive disorder, such as major depression, minor depression, or loneliness feelings [21]. Physicians mostly check for other related clinical features of hypothyroidism to distinguish hypothyroidism-related mood disturbance from other common sources of a persistently depressed mood [22]. There is clearly a link between thyroid problems and psycho-social disorders, according to an extensive study review published in 2012 [23].

The current study revealed that 2.2% and 16% of the studied hypothyroid patients had moderately high and high degrees of loneliness feelings, respectively. This high level of loneliness feelings had a non-significant relationship with patients’ age, genders, education, SES, smoking, or practicing physical activity.

The only significant factor associated with a high level of loneliness feeling was hypothyroidism duration rather than thyroid profile (TSH, T3, and T4). This can be explained by the fact that hypothyroidism requires time to manifest in mood changes or psychological upsets and is difficult to examine with a laboratory profile. Correlation analysis showed a borderline significant relation between T3 level and the feeling of loneliness score. Hypothyroidism is one of the metabolic disorders that is associated with psychological disturbances among cases, including depression. Depressed patients lose all luxury desires and have a high tendency toward isolation, which results in loneliness preference [24,25].

Demartini et al., [24] found that 63.4% of hypothyroid patients had a diagnosis of depressive episodes with anxiety. Demartini et al., [25] reported that 63.5% of hypothyroid patients had psychological disorders. The most frequent symptoms were anxiety and somatization, cognitive impairment disturbances, psychomotor retardation, and sleep disorders, which all may end in social isolation and loneliness feelings among patients. Guimarães et al., [26] estimated that 45.7% of hypothyroid patients presented with depressive symptoms. Females with a TSH > 10 mUI/ml had a threefold likelihood of presenting depressive symptoms compared to those with normal levels of TSH. Among those with clinical hypothyroidism, the adjusted OR was 8.7. In a wider context, many studies have focused on the relationship between loneliness with social isolation and chronic diseases in general. There has been no consensus regarding the nature and significance of this relation, as some studies have confirmed this relation but other studies have failed to find any such association [27].

Limitations

Our study has some limitations. First, in our opinion, an important limitation was that the use of a self-administered questionnaire could have a recall bias. Second, the majority of our study sample was female so our study did not include undiagnosed mood disorders, menstrual cycle changes, and presence/absence of social or familial support.

## Conclusions

In conclusion, the study revealed that 2.2% and 16% of the sampled hypothyroid patients had moderately high and high degrees of loneliness feelings, respectively. The only significant predictor for high loneliness score was hypothyroidism duration rather than thyroid profile or other patient-related factors.

## Appendices

Questionnaire
 

1- Marital status: 

a. Married 

b. Single

c. Divorced 

d. Widow
 

2- Age: In years
 

3- Gender:

a. Male

b. Female
 

4- Level of education:

a. None

b. High secondary school or less

c. Bachelor's degree

d. Master degree

5- Socioeconomic status:

a. Low (less than 5000 SR)

b. Medium (5000-15,000)

c. High (more than 15,000 SR)
 

6- Smoking:

a. Yes (if yes for how long, which type)

b. No

c. Ex-smoker

d. Passive smoker

7- Do you live alone?

a. Yes

b. No

8- Do you have any chronic diseases?

a. No

b. Yes (diabetes)

c. Yes (hypertension)

9- Duration of hypothyroidism: in years
 

10-Medication use (levothyroxine)

a.yes

b.no

11- Dose of levothyroxine? (...)

12- Physical activity

a. Sedentary lifestyle

b. <150 minutes\week

c. Around 150-300 minutes\week

d. >300 minutes\week

13- Do you live alone? 

a.Yes

b.No
 

14- Hight (cm)

15- Weight (kg)

**Table 4:** Loneliness score

Statement	Never	Rarely	Sometimes	Often
1- I feel in tune with the people around me	1	2	3	4
2- I lack companionship	1	2	3	4
3- There is no one I can turn to	1	2	3	4
4- I do not feel alone	1	2	3	4
5- I feel part of a group of friends	1	2	3	4
6- I have a lot in common with the people around me	1	2	3	4
7- I am no longer close to anyone	1	2	3	4
8- My interests and ideas are not shared by those around me	1	2	3	4
9- I am an outgoing person	1	2	3	4
10- There are people I feel close to	1	2	3	4
11- I feel left out	1	2	3	4
12- My social relationships arc superficial	1	2	3	4
13- No one really knows me well	1	2	3	4
14- I feel isolated from others	1	2	3	4
15- I can find companionship when I want it	1	2	3	4
16- There are people who really understand me	1	2	3	4
17- I am unhappy being so withdrawn	1	2	3	4
18- People are around me but not with me	1	2	3	4
19- There are people I can talk to	1	2	3	4
20- There are people I can turn to	1	2	3	4
